# The Halophyte Species *Solanum chilense* Dun. Maintains Its Reproduction despite Sodium Accumulation in Its Floral Organs

**DOI:** 10.3390/plants11050672

**Published:** 2022-02-28

**Authors:** Servane Bigot, Paula Pongrac, Martin Šala, Johannes T. van Elteren, Juan-Pablo Martínez, Stanley Lutts, Muriel Quinet

**Affiliations:** 1 Groupe de Recherche en Physiologie Végétale (GRPV), Earth and Life Institute-Agronomy (ELI-A), Université Catholique de Louvain, Croix du Sud 4-5, 1348 Louvain-la-Neuve, Belgium; stanley.lutts@uclouvain.be (S.L.); muriel.quinet@uclouvain.be (M.Q.); 2 Department of Biology, Biotechnical Faculty, University of Ljubljana, Večna Pot 111, 1000 Ljubljana, Slovenia; paula.pongrac@bf.uni-lj.si; 3 Department of Analytical Chemistry, National Institute of Chemistry, Hajdrihova 19, 1000 Ljubljana, Slovenia; Martin.Sala@ki.si (M.Š.); elteren@ki.si (J.T.v.E.); 4 Instituto de Investigaciones Agropecuarias (INIA-La Cruz), Chorrillos 86, La Cruz 2280454, Chile; jpmartinez@inia.cl

**Keywords:** salinity, inflorescences, ion localization

## Abstract

Salinity is a growing global concern that affects the yield of crop species, including tomato (*Solanum lycopersicum*). Its wild relative *Solanum chilense* was reported to have halophyte properties. We compared salt resistance of both species during the reproductive phase, with a special focus on sodium localization in the flowers. Plants were exposed to NaCl from the seedling stage. Salinity decreased the number of inflorescences in both species but the number of flowers per inflorescence and sepal length only in *S. lycopersicum*. External salt supply decreased the stamen length in *S. chilense*, and it was associated with a decrease in pollen production and an increase in pollen viability. Although the fruit set was not affected by salinity, fruit weight and size decreased in *S. lycopersicum*. Concentrations and localization of Na, K, Mg, and Ca differed in reproductive structures of both species. Inflorescences and fruits of *S. chilense* accumulated more Na than *S. lycopersicum*. Sodium was mainly located in male floral organs of *S. chilense* but in non-reproductive floral organs in *S. lycopersicum*. The expression of Na transporter genes differed in flowers of both species. Overall, our results indicated that *S. chilense* was more salt-resistant than *S. lycopersicum* during the reproductive phase and that differences could be partly related to dissimilarities in element distribution and transport in flowers.

## 1. Introduction

Tomato (*Solanum lycopersicum*) is cultivated worldwide and is of great economic importance. In 2020, more than 6 Mha of tomato plants was cultivated and 252 Mt of fruits was harvested [1]. Plant breeding increased tomato yields, and the world average yield in 2020 was 598 t ha^−1^ with values ranging from 14 t to 5 kt ha^−1^, depending on the region and the cultural mode [1]. However, tomato is sensitive to abiotic stresses, including salinity, because of its glycophytic nature [2]. Salinity is a growing global concern, and it is estimated that salinity is present in 900 million ha of soils worldwide [3]. Sodium chloride (NaCl) is the most common of salts and represents more than 90% of salt in the world [4]. Tomato is cultivated in many countries affected by salinity (e.g., East Asia, the Middle East, and North Africa), and salinity decreases tomato yield by on average 50% for an electrical conductivity of 5 dS m^−1^ [5].

Despite decades of tomato breeding programs, resistance to abiotic stress has been neglected [6]. Indeed, since the 1960s, tomato improvement has mainly focused on fruit yield, shelf-life, and taste [7,8]. Because of the self-pollination of cultivated tomato and varietal selection, genetic diversity has been considerably lost in this species [9]. Miller and Tanksley [10] estimated that the *S. lycopersicum* genome contained less than 5% of the genetic variation of its wild relatives and, according to Bretó et al. [11], this species is considered to have the lowest genetic diversity in the tomato clade (Clade II of *Solanum*, consisting of *S. lycopersicum*, *S. tuberosum*, and *S. muricatum*, [12]). *Solanum lycopersicum* has many wild relatives including a few originating from harsh environments [13]. The use of resistant wild relatives in breeding is a common practice to improve the resistance of crop species to abiotic stresses [14]. *Solanum chilense* is a wild tomato relative native from the Atacama desert, one of the most salty and arid areas in the world [15,16]. Due to its high level of genetic variability, *S. chilense* is considered one of the most promising sources of genes for selection of tomato genotypes resistant to abiotic and biotic stress [11,17,18]. Like some tomato relatives, *S. chilense* is self-incompatible and requires cross-pollination, while *S. lycopersicum* is self-compatible and self-pollinates [16]. The resistance of *S. chilense* to biotic stress has been largely investigated, and this species has been used in breeding programs for resistance to viruses such as the tomato yellow leaf curl virus [19] or the cucumber mosaic virus [20]. However, despite a great interest in improving the abiotic stress resistance of tomato, investigation into the resistance of *S. chilense* to abiotic stress such as salinity is rarely studied [2,21].

The effects of NaCl stress on *S. lycopersicum* culture have been explored for a long time, and studies have mainly focused on vegetative growth or yield parameters [8,21,22,23]. Even if fruit formation is a direct function of reproduction efficiency, the flowering stage is a necessary process before fructification and is consequently impacted by salinity stress before fruit formation. However, the effect of salt on reproductive structures has been little explored in tomato, although abiotic stresses and more specifically salinity may have an impact on the flowering stage. The reproductive phase is indeed considered one of the most sensitive plant developmental stages toward salinity [24]. Ultimately, salinity leads to a decrease in fruit yield and fruit weight and modification of sugar concentration and antioxidant compounds [25,26]. However, earlier in the reproductive development, it can lead to decrease of flower production or decrease of pollen germination and pollen tube growth and even modifications of flower morphology [24,27,28]. In tomato, salinity was shown to induce inflorescence failure and fertility decrease [29,30]. Nevertheless, how salinity affects the flowering and reproductive stage of the halophyte *S. chilense* remains largely unknown.

*Solanum chilense* has been shown to accumulate more Na in the vegetative aerial parts than *S. lycopersicum* in response to salt [2] but Na accumulation in the reproductive parts has not been investigated as yet. Sodium transport and storage play key roles in the plant response to salinity [31]. Transporters of mineral elements involved in salinity resistance have been widely studied in several plant species, including tomato [32,33]. Several families of transporters are indeed involved in salinity resistance at different stages, especially to maintain Na and potassium (K) homeostasis [34,35]. Briefly, sodium can enter the cell via class I-HKT (High Affinity K^+^) transporters and non-selective cation channels. Other transporters, such as the SOS (salt overly sensitive) pathway genes are involved in Na exclusion [34,35,36]. NHX (vacuolar Na^+^/H^+^ antiporters) transporters are believed to be Na^+^/H^+^ exchangers implied in vacuolar Na^+^ sequestration [37,38]. Other transporters may play a role in salinity resistance in other ways. HAK (High Affinity K^+^) transporters are involved in potassium nutrition and so could help against salt stress [39]. AKT2/3 (inward-rectifying K^+^ channel) is a potassium transporter involved in sucrose import in the phloem, which is also activated in response to salt stress [40,41]. In inflorescences of tomato, silencing of HKT1;2 was shown to increase the Na^+^/K^+^ ratio [25]. However, involvement of transporters activity in salinity resistance in the reproductive structures remains largely unknown in tomato.

In this paper, we compared the Na and K concentrations and localization in the reproductive structures of the halophyte *S. chilense* and the glycophyte *S. lycopersicum* as affected by salt stress and investigated responses of the reproduction of *S. chilense* to salt stress. We aimed to answer the following questions: (1) How does salinity affect flowering, flower development and fertility, and fruit production in these species? (2) Does salinity affect Na and mineral accumulation and partitioning in the reproductive structures of the two species? (3) Does a different Na partitioning in flowers affect flower fertility? (4) What are the responses of putative Na transporters and their contribution to Na accumulation and partitioning in the reproductive structures?

## 2. Results

### 2.1. Impact of Salinity on Reproductive Growth

**Figure 1:**
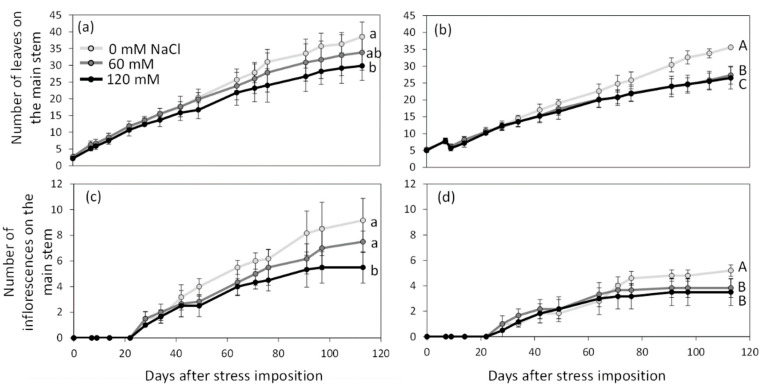
Number of leaves (**a**,**b**) and of inflorescences (**c**,**d**) on the main stem of *Solanum lycopersicum* (**a**,**c**) and *Solanum chilense* (**b**,**d**) grown in perlite:vermiculite mixture supplied with 0, 60, and 120 mM NaCl from 0 to 113 days after stress imposition. Data are means ± SD, treatments followed by different letters are significantly different (lowercase, *S. lycopersicum*, uppercase, *S. chilense*) at *p* < 0.05 for a same species at 113 DASt.

Salt stress was applied before floral transition up to fruit maturation. Throughout the experiment, *S. lycopersicum* produced more leaves on the main stem than *S. chilense*, even under salt stress conditions (Figure 1a,b, Table S2). At 113 days after stress imposition (DASt), the average number of leaves on the main stem was 34.06 ± 5.72 in *S. lycopersicum* and 29.47 ± 4.77 in *S. chilense* (Figure 1a,b). Salt decreased the leaf production in both species (Figure 1a,b): leaf production decreased gradually with stress intensity in *S. lycopersicum* while it was similar in plants treated with 60 and 120 mM NaCl in *S. chilense*. As *S. chilense* had a bushier appearance than *S. lycopersicum*, the total number of leaves produced at 85 DASt was higher in *S. chilense* (80.22 ± 46.63) than in *S. lycopersicum* (47.67 ± 29.97) but it also decreased by 71% and 65% with salt stress, respectively.

Regarding reproductive growth, flowering times of the initial and sympodial segments were similar between species and salt treatments (Table 1 and Table S2). However, as observed for leaf production, *S. lycopersicum* produced more inflorescences on the main stem than *S. chilense* (Figure 1c,d): at 113 DASt, 7.39 ± 1.97 and 4.12 ± 1.05 inflorescences were observed on the main stem of *S. lycopersicum* and *S. chilense*, respectively. Taking into account the ramifications, the total number of inflorescences per plant was similar in both species (Table 1 and Table S2). NaCl decreased the number of inflorescences on the main stem and the total number of inflorescences per plant in both species (Table 1 and Table S2); the effect was dose-dependent in *S. lycopersicum* but not in *S. chilense* (Figure 1c,d, Table 1). The number of floral buds per inflorescence was always higher in *S. chilense* than in *S. lycopersicum* (Table 1 and Table S2). This number decreased with salt stress in *S. lycopersicum* but not in *S. chilense*. In the same way, salinity decreased the percentage of flower buds reaching anthesis only in the cultivated tomato (Table 1 and Table S2).

### 2.2. Impact of Salinity on Flower Morphology and Fertility

Flower morphology differed among tomato species (Table 2 and Table S2): sepals, petals, and stamens were always longer in *S. lycopersicum* than in *S. chilense*, while pistils were longer in *S. chilense* than in *S. lycopersicum* and style exertion was only observed in *S. chilense*. Salt affected flower morphology by decreasing the length of sepals in *S. lycopersicum* and modifying the length of stamens in *S. chilense*.

Flower fertility was assessed by stigma receptivity, pollen production, and viability (Table 2 and Table S2). Overall, stigma receptivity was slightly lower in *S. lycopersicum* than in *S. chilense*. *S. lycopersicum* also produced fewer pollen grains per anther than *S. chilense*. However, pollen viability was 23% higher in *S. lycopersicum* than in *S. chilense*. Salt did not affect stigma receptivity, pollen viability, or the number of pollen grains per anther in *S. lycopersicum*. However, in *S. chilense*, the number of pollen grains per anther decreased with salt while pollen viability increased gradually with salt concentration.

### 2.3. Impact of Salinity on Fruit Production and Quality

Fruit set was higher in *S. chilense* than in *S. lycopersicum* and was not affected by salt stress whatever the species (Table 3 and Table S2).

*S. lycopersicum* produced bigger fruits than *S. chilense*. Indeed, fruit FW, DW, WC, and size were higher in *S. lycopersicum* than in *S. chilense* (Table 3). Following the fruit size, the number of seeds per fruit was 69% higher in *S. lycopersicum* than in *S. chilense* (Table 3), although, when expressed per gram of fruit FW, the number of seeds was 90% higher in *S. chilense* than in *S. lycopersicum*. Salinity mainly affected fruit growth in *S. lycopersicum* as fruit DW, FW, WC, and size decreased with a higher salt concentration in *S. lycopersicum* while salinity modified only fruit WC in *S. chilense*, which increased with salt concentration (Table 3). However, the number of seeds per fruit or per gram of fruit FW were not affected by salinity whatever the species (Table 3).

Concerning fruit quality, fruits of *S. lycopersicum* were less sweet and less acidic than those of *S. chilense* (Table 3): sugar content and pH were, respectively, 3.3 and 1.1 times lower in fruits of *S. lycopersicum* than in the ones of *S. chilense* under control conditions. Salinity affected fruit quality in both species (Table S2). The fruit sugar content was modified in different ways according to the species: sugar concentration increased in *S. lycopersicum* but decreased in *S. chilense* with salt concentration (Table 3). However, fruit pH decreased with salinity in both species (Table 3).

### 2.4. Impact of Salinity on Mineral Concentration and Distribution in Reproductive Organs

#### 2.4.1. Inflorescences and Flowers

**Figure 2:**
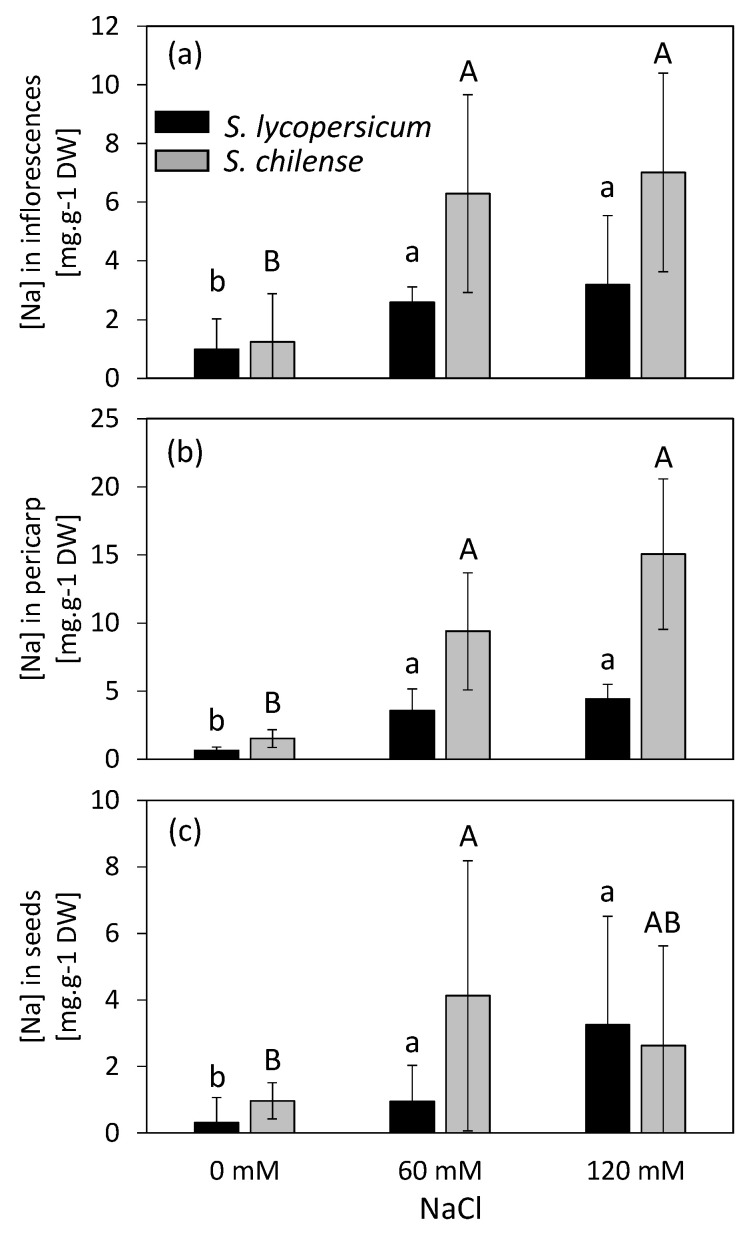
Sodium (Na) concentration in inflorescences (**a**), pericarp of fruits (**b**), and seeds (**c**) of *Solanum lycopersicum* and *Solanum chilense* grown in perlite:vermiculite mixture supplied with 0, 60, and 120 mM NaCl. Data are means ± SD; treatments followed by different letters are significantly different (lowercase, *S. lycopersicum*, uppercase, *S. chilense*) at *p* < 0.05 for a same species.

**Figure 3:**
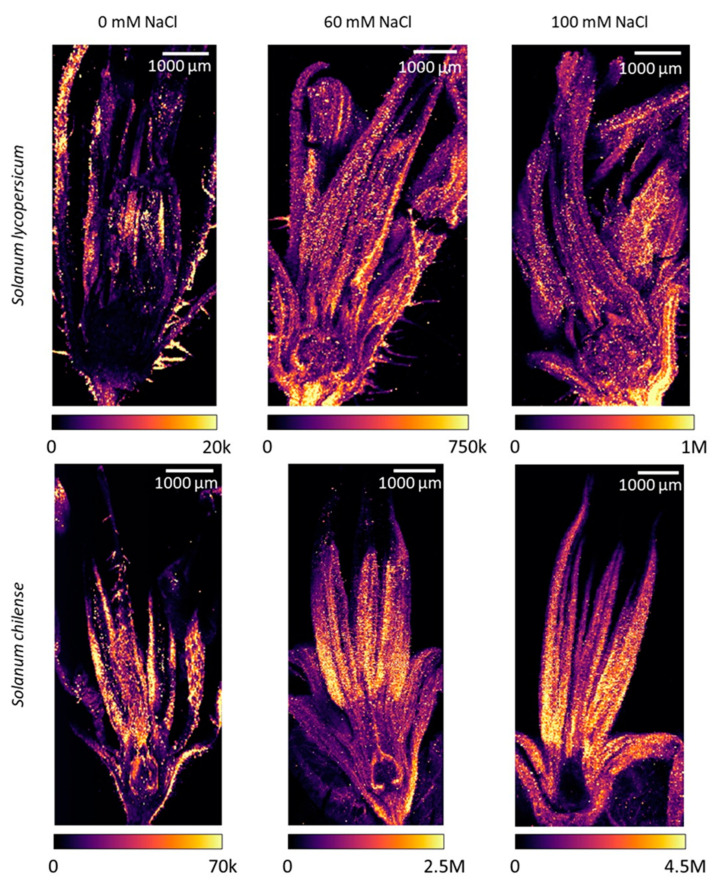
Sodium (Na) distribution in flowers of *Solanum lycopersicum* (top row) *and Solanum chilense* (bottom row) grown in perlite:vermiculite mixture supplied with 0, 60, and 100 mM NaCl as revealed by LA-ICP-MS (laser ablation inductively coupled plasma mass spectroscopy) and visualized using ImageJ (version 1.53a). Color legends represent the number of counts per pixel (20 × 20 µm^2^) of each analysis.

Inflorescences of *S. chilense* accumulated more Na than the ones of *S. lycopersicum* (Figure 2a), even under control conditions. Salinity induced a significant increase in Na concentration in the inflorescences of both species (Figure 2a, Table S2), although it was larger in *S. chilense* than in *S. lycopersicum*. Indeed, Na concentration increased by 223% and 465%, given as the percentual difference between control and 120 mM NaCl treated plants in *S. lycopersicum* and *S. chilense*, respectively. Moreover, Na distribution mapping showed that, in addition to the Na concentration, there was a difference in Na location inside the flowers in the two species (Figure 3 and Figure S1). In *S. chilense*, Na mainly accumulated in the male organs (Figure 3), whereas in *S. lycopersicum*, most of the Na was located in the receptacle and pedicel (Figure 3). Moreover, the ratio between the number of counts of Na in the floral receptacle and reproductive (stamens + pistil) floral whorls was higher in *S. lycopersicum* than in *S. chilense* and increased with salt stress, mainly in *S. lycopersicum* (Table 4). The ovary had the lowest Na signal compared to the rest of the flower in both species (Figure 3). As a result, the ratio between the Na signal in the stamens and the pistil was higher in *S. chilense* than in *S. lycopersicum* (Table 4). This ratio decreased with salt stress in both species.

**Figure 4:**
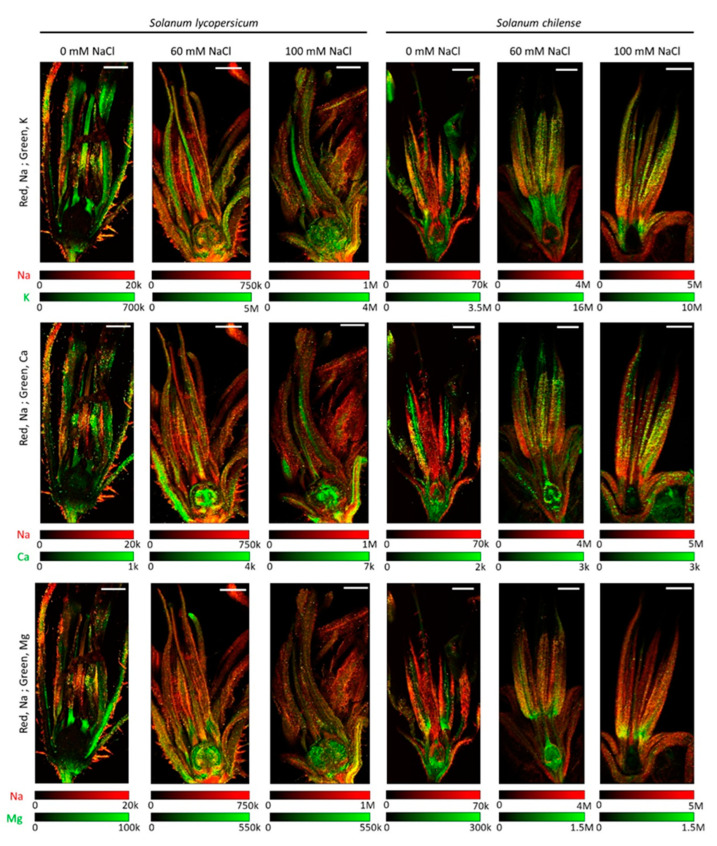
Distribution of sodium (Na) shown in red and potassium (K), calcium (Ca) and magnesium (Mg) shown in green and their co-localization (yellow) in flowers of *Solanum lycopersicum* and *Solanum chilense* grown in perlite:vermiculite mixture supplied with 0, 60, and 100 mM NaCl. Distribution of individual element was determined using LA-ICP-MS (laser ablation inductively coupled plasma mass spectroscopy) and visualized using ImageJ (version 1.53a). Color legends represent the number of counts per pixel (20 × 20 µm²) for each analysis and each element. Signal intensities are correlated with the concentrations of a particular element. Scale bar = 1000 µm.

As for Na, inflorescences of *S. chilense* accumulated more K than those of *S. lycopersicum* (Table 5 and Table S2). Salinity did not affect the K concentration in the inflorescences whatever the species (Table 5 and Table S2). The K/Na ratio was, however, higher in the inflorescences of *S. lycopersicum* than in those of *S. chilense* and decreased with salt stress in both species (Table 5 and Table S2). In flowers of *S. chilense*, K mainly accumulated in male organs with no accumulation in female organs (Figure 4 and Figure S2). In contrast, K accumulated mainly in female organs in *S. lycopersicum* (Figure 4 and Figure S2). As a result, the ratio of the number of counts of K in stamens and pistil was higher in *S. chilense* than in *S. lycopersicum* (Table 4). However, the ratio between the K signals in floral receptacle and reproductive floral organs was similar in both species under control conditions but decreased with salt in *S. chilense* and not in *S. lycopersicum* (Table 4).

Inflorescences of *S. lycopersicum* accumulated about 10 times more Ca than those of *S. chilense*, and their Ca concentrations were not affected by salinity (Table 5 and Table S2). Ca mainly accumulated in floral receptacle of *S. lycopersicum* and mainly in reproductive floral organs of *S. chilense* (Figure 4 and Figure S3). Indeed, the ratio between the Ca signals in floral receptacle and reproductive floral organs was higher in *S. lycopersicum* than in *S. chilense* (Table 4). Ca was particularly visible in the ovary of salt-treated *S. lycopersicum* flowers (Figure 4 and Figure S3), explaining the lower ratio of Ca signal between stamens and pistil in salt-treated flowers (Table 4).

The concentration of Mg in inflorescences of *S. lycopersicum* was more important than in those of *S. chilense* (Table 5 and Table S2). However, only the former was affected by salinity (Table 5 and Table S2). Mg mainly accumulated in the stamens and ovary of *S. chilense* and in the ovary of *S. lycopersicum* (Figure 4 and Figure S4). The ratio of Mg signals between floral receptacle and reproductive floral organs and between stamens and pistil decreased with salt stress in *S. chilense* and *S. lycopersicum*, respectively (Table 4).

#### 2.4.2. Fruits and Seeds

The Na concentration in fruit pericarp was similar to in the inflorescences for the same species (*S. lycopersicum*, t_101_ = −0.161, *p* = 0.872, *S. chilense*, t_49_ = −0.818, *p* = 0.417, Figure 2a,b). Nevertheless, as observed in the inflorescences, the pericarp of *S. lycopersicum* fruits were less concentrated in Na than the pericarp of *S. chilense* fruits (Figure 2b, Table S2): the difference was about 2.4 times that of control plants, 2.6 times that of 60 mM NaCl treated plants, and 3.4 times that of 120 mM NaCl treated plants. Salinity indeed increased the Na concentration in the pericarp of both species but to a higher extent in *S. chilense*. For both species, the Na concentration was 0.6 and 0.4 times lower in seeds than in pericarp for *S. lycopersicum* and *S. chilense*, respectively, but again, seeds of *S. chilense* contained more Na that the ones of *S. lycopersicum* (Figure 2c, Table S2). However, the Na concentration increased with salt stress in the seeds of *S. lycopersicum*, but only slightly in those of *S. chilense* (Figure 2b,c).

The concentrateion of K in the pericarp was similar in both species and decreased significantly with salt stress in both species (Table 5 and Table S2). However, the K concentration in the seeds was higher under control conditions in *S. chilense* than in *S. lycopersicum*, and it decreased with salinity only in the former so that the K concentration was similar in the seeds of stressed plants of both species (Table 5 and Table S2).

The concentration of Ca was higher in the pericarp of *S. chilense* than in the one of *S. lycopersicum*, but there was no clear difference under salinity (Table 5 and Table S2). However, the Ca concentration in seeds did not differ between species (Table 5 and Table S2). The concentration of Mg was higher in the pericarp of *S. chilense* than in the one of *S. lycopersicum*, but it was higher in the seeds of *S. lycopersicum* than in the ones of *S. chilense* (Table 5 and Table S2).

### 2.5. Impact of Salinity on the Expression of Mineral Transporters in Flowers

To improve our understanding of Na accumulation and its distribution in flowers, we investigated the expression of genes coding for transporters involved in Na transport in flowers at anthesis. We particularly focused on the SOS pathway, and the NHX, HKT and HAK transporters.

**Figure 5:**
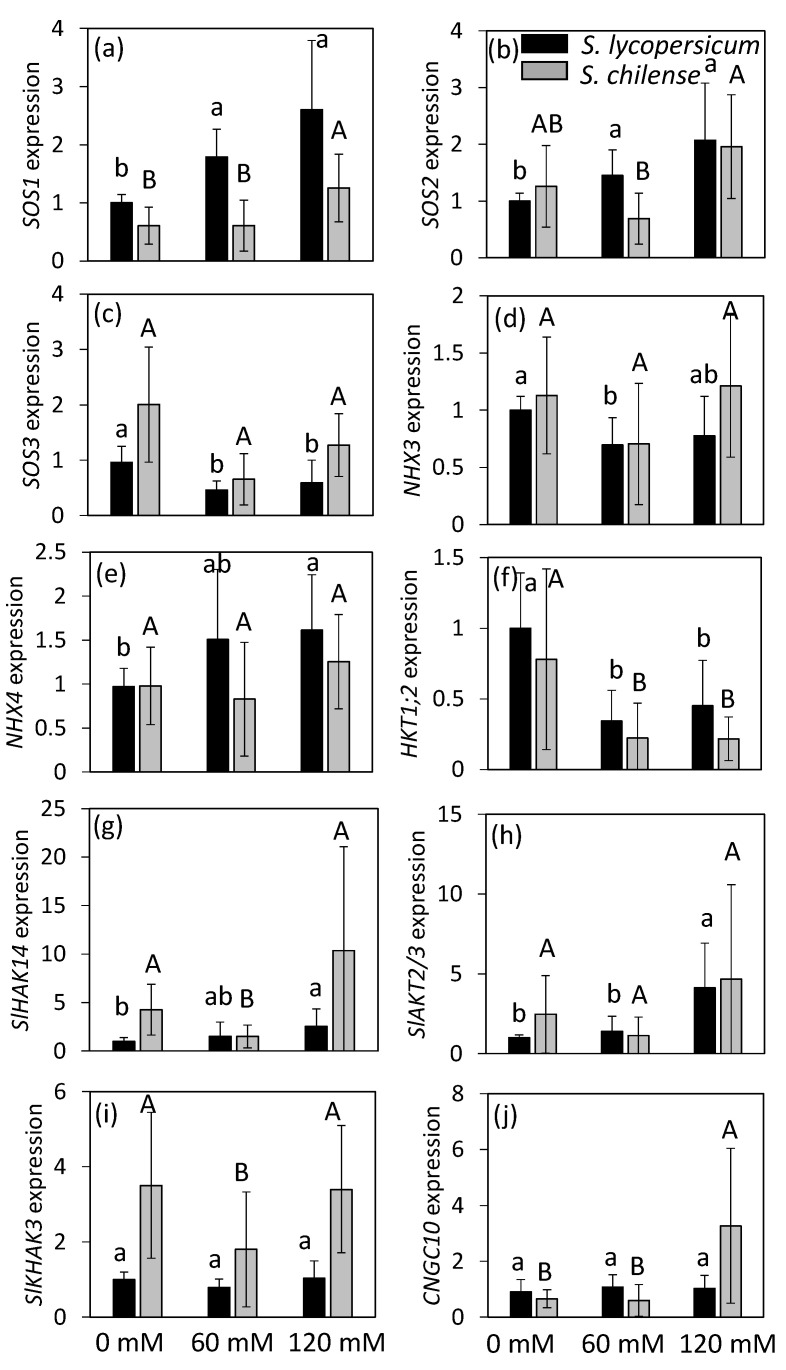
Expression of 10 genes involved in minerals transport analyzed by qRT-PCR on flowers of *Solanum lycopersicum* and *Solanum chilense* growing at 0, 60, and 120 mM NaCl. (**a**) *SOS1* (Salt Overly Sensitive 1, Solyc01g005020); (**b**) *SOS2* (Salt Overly Sensitive 2, Solyc12g009570); (**c**) *SOS3* (Salt Overly Sensitive 3, Solyc06g051970); (**d**) *NHX3* (vacuolar Na^+^/H^+^ antiporter 3, Solyc01g067710); (**e**) *NHX4* (vacuolar Na^+^/H^+^ antiporter 4, Solyc01g098190); (**f**) *HKT1;2* (class I—High affinity K^+^ transporter 2, Solyc07g014680); (**g**) *SlHAK14* (High Affinity K^+^ transporter 14, Solyc09g074820); (**h**) *SlAKT2/3* (inward-rectifying K^+^ channel, Solyc10g024360); (**i**) *SlHAK3* (High Affinity K^+^ transporter 3, Solyc12g096580); (**j**) *CNGC10* (Cyclic Nucleotide Gated Channel 10, Solyc05g050350). The tomato elongation factor gene (*LeEF-1α*, Solyc06g005060) and TIP41-like protein (*TIP41*, Solyc10g04985) were used as the reference genes. Expressions are given based on *S. lycopersicum* grown at 0 mM NaCl, to which a value of 1 was assigned. Data are means ± SD, treatments followed by different letters are significantly different at *p* < 0.05 for the same species (lowercase, *S. lycopersicum*, uppercase, *S. chilense*).

Concerning the SOS pathway, *SOS1* expression was higher in *S. lycopersicum* than in *S. chilense*, while the opposite trend was observed for *SOS3* expression (Figure 5a,c, Table S2). However, there was no difference of expression for *SOS2* between species (Figure 5b, Table S2). Salt stress increased *SOS1* expression in both species but more significantly and at a lower salt concentration in *S. lycopersicum* than in *S. chilense* (Figure 5a). Expression of *SOS2* and *SOS3*, respectively, increased and decreased with salt in *S. lycopersicum* only; nevertheless, a decrease of *SOS3* expression was observed in *S. chilense* at 60 mM NaCl (Figure 5b,c).

The gene *NHX3*, which encodes a tonoplast transporter, had similar expression levels in both species regardless of treatment (Figure 5d, Table S2), contrary to *NHX4*, which was more expressed in *S. lycopersicum* than in *S. chilense* at least in salt-treated flowers (Figure 5e, Table S2). Salt stress decreased the expression of *NHX3* and increased the expression of *NHX4* in *S. lycopersicum* but did not affect their expression in *S. chilense* (Figure 5d,e).

The expression of *HKT1;2* was slightly higher in *S. lycopersicum* than in *S. chilense* and decreased with salt treatment in both species from 60 mM NaCl (Figure 5f, Table S2).

The expression of *SlHAK14* and *SlHAK3* was higher in *S. chilense* than in *S. lycopersicum*, while the expression of *SlAKT2/3* and *CNGC10* was similar in both species (Figure 5g–j, Table S2). Salinity affected these genes differently, depending on the species. The expression of *SlHAK14* gradually increased with salt in *S. lycopersicum* but decreased in *S. chilense* at 60 mM NaCl only (Figure 5g). The expression of *SlAKT2/3* increased in *S. lycopersicum* from 60 mM NaCl but was unchanged in *S. chilense* (Figure 5h). The expression of *SlHAK3* was stable in *S. lycopersicum* but decreased at 60 mM NaCl in *S. chilense* (Figure 5i). The expression of *CNGC10* was stable in *S. lycopersicum* but increased at 120 mM NaCl in *S. chilense* (Figure 5j).

### 2.6. Correlations among Flower Morphology, Mineral Concentrations, and Gene Expression

**Figure 6:**
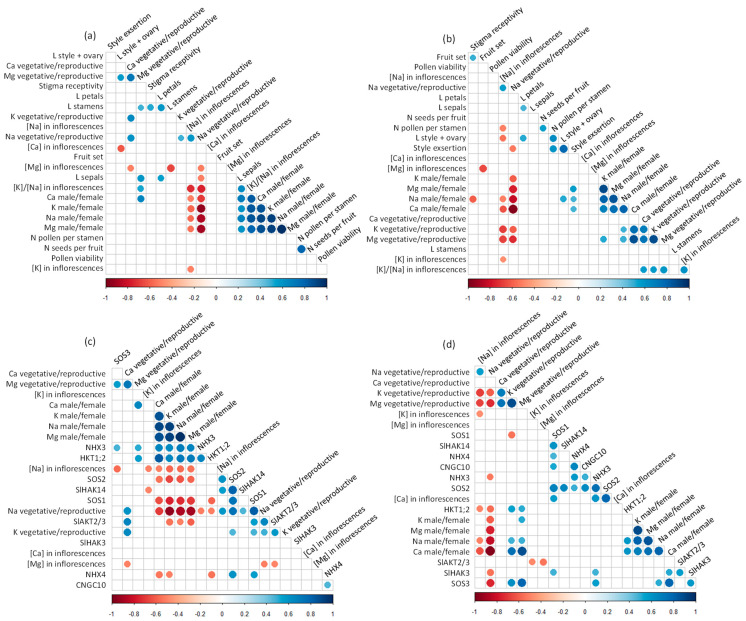
(**a**,**b**) Correlation graphs of concentrations of elements in inflorescences, ratios of element signals in the vegetative/reproductive organs and male/female organs on flowers and fertility parameters of flowers of *Solanum lycopersicum* (**a**) and *Solanum chilense* (**b**). (**c**,**d**) Correlation graphs of concentrations of elements in inflorescences, ratios of element signals in the vegetative/reproductive organs and male/female organs on flowers and expression of mineral transporters in flowers of *S. lycopersicum* (**c**) and *S. chilense* (**d**). Only significant correlations (*p* < 0.05) are indicated with circles. Negative correlations are highlighted in red and positive correlations in blue. *CNGC10*, Cyclic Nucleotide Gated Channel 10; *HKT1;2*, class I—High affinity K^+^ transporter 2; L petals, sepals, style + ovary: length of, respectively, petals, sepals, and the sum of style and the ovary; *NHX3*, *4*, vacuolar Na^+^/H^+^ antiporter 3, 4; N pollen per stamen, seeds per fruit: number of, respectively, pollen grains per stamen and seeds per fruit; *SlAKT2/3*, inward-rectifying K^+^ channel; *SlHAK3*, *14*, High Affinity K^+^ transporter 3,14; *SOS1*, *2*, *3*, Salt Overly Sensitive 1, 2, 3.

Analysis of correlations among flower fertility parameters, concentrations of elements in inflorescences and flowers, and expression of mineral transporters in flowers showed a different behavior between both species (Figure 6). Overall, few correlations were observed between flower fertility parameters and mineral concentrations in the flowers, mainly in *S. lycopersicum* (Figure 6a,b). In *S. chilense*, the number of pollen grains per stamen was negatively correlated with the concentration of Na in inflorescences, although this correlation was not observed in *S. lycopersicum*. Some correlations were observed between floral organ size and elements signals in the reproductive structures in both species (Figure 6a,b). In *S. lycopersicum*, sepal length was negatively correlated with the ratio of Na signal between vegetative and reproductive floral organs and positively correlated with the ratio of elements signals between male and female reproductive organs and with the K/Na ratio in the inflorescences (Figure 6a). Moreover, in *S. chilense*, the pistil length and the style exertion were negatively correlated with, respectively, the Na concentration in the inflorescence and the ratio of Na signal between vegetative and reproductive organs (Figure 6b). Stamen and pistil lengths were also negatively correlated with, respectively, the Ca and Mg concentrations in inflorescences and positively correlated with the ratio of Mg signals between vegetative and reproductive floral organs in *S. lycopersicum*. Correlations between Na signals in reproductive structures and Na transporter gene expression also differed among species (Figure 6c,d). The Na concentration in inflorescences was negatively correlated with the expression of *SOS3* and positively correlated with the expression of *SOS2* and *SlHAK14* in *S. lycopersicum* while it was negatively correlated with the expression of *HKT1;2* in *S. chilense* (Figure 6c,d). The ratio of Na concentrations in male and female floral organs was negatively correlated with the expression of *SOS1*, *SOS2*, and *SlAKT2/3* and positively correlated with the expression of *NHX3* and *HKT1;2* in *S. lycopersicum* while it was positively correlated with the expression of *SOS3* and *HKT1;2* in *S. chilense*. The ratio between Na signals in vegetative and reproductive floral organs was negatively correlated with the expression of *NHX3* and *HKT1;2* in both species; it was also negatively correlated with the expression of *SlHAK3* and *SOS3* in *S. chilense* and positively correlated with the expression of *SOS1*, *SOS2*, *SlHAK14*, and *SlAKT2/3* in *S. lycopersicum* (Figure 6c,d).

## 3. Discussion

### 3.1. Salinity Affects Reproductive Structures in Both Species

Flowering and reproduction differed between *S. lycopersicum* and *S. chilense*. The former is considered as an autonomous flowering plant [42] while the latter is a short-day plant [16,43]. Moreover, *S. lycopersicum* is self-compatible and self-pollinates while *S. chilense* is self-incompatible and requires insect pollination [13]. We observed that salinity affected the reproductive phase in both species but in different ways. Salt stress decreased the number of inflorescences in both species but the number of floral buds and opened flowers per inflorescence was only reduced in *S. lycopersicum*. *Solanum chilense* produced more flowers per inflorescence than *S. lycopersicum* like most wild tomato relatives, which could be an advantage for breeding [44], but this parameter was not affected by salt stress in *S. chilense*. Inflorescence and flower production seemed thus more affected by salinity in *S. lycopersicum* than in *S. chilense*, and the effect was more dose-dependent in the former than in the latter. Flower abortion was previously observed under salt conditions in cultivated tomato [29]. A decrease in inflorescence and flower production and an increase in flower abortion are common phenomena observed in response to stress; abortion of spikelets was, for instance, observed in rice under salinity treatments [45].

Salinity also affected flower morphology and fertility. Flower morphology differed between species: the ratio between corolla and calyx area was higher in *S. chilense* than in *S. lycopersicum*, and style exertion was observed only in the former. These differences could be related to the self-incompatibility of *S. chilense* [16] that needs to attract pollinators for cross-pollination. Concerning floral organs, salt decreased sepal length in *S. lycopersicum* and decreased stamen length in *S. chilense*. Modification of flower morphology due to salinity was reported in *Spergularia maritima* (petal size increased in salinity treatments) [27]. In tomato, other environmental constraints such as temperature also affect flower morphology [46,47]. Those modifications could have an impact on flower attractivity for pollinators, as it has been shown in *Raphanus sativus* [48] or *Borago officinalis* [49,50]. Flower and petal size are indeed important floral signals for pollinators [49]. The decrease of stamen length observed in salt-treated *S. chilense* was associated with a decrease in the number of pollen grain per anther and an increase in pollen viability. However, in our study, pollen production and viability were not affected by salinity in *S. lycopersicum*, and stigma receptivity was not affected by salt stress whatever the species. Anther development and microsporogenesis are generally considered the most sensitive reproductive stages to abiotic stresses, which could explain the more important effect on male organs than on female organs [51]. Gynoecium fertility is not often affected by abiotic stress in tomato or is affected as a consequence of male development failure [52,53].

In accordance with the low impact of salinity on flower fertility, fruit set was not affected by salt treatment in our study whatever the species. However, fruit weight, size, and water content decreased with salinity in *S. lycopersicum* while these parameters were not affected or even increased (for WC) under salt treatment in *S. chilense*. Moreover, the seed set decreased with salinity in *S. lycopersicum* but not in *S. chilense*. The effect of salinity on flower fertility is thus not sufficient to explain the salt-induced modifications of fruit parameters despite the positive correlation between pollen per anther and seeds per fruit. Pollen tube growth, fertilization, and seed development may be affected by abiotic stress such as salinity [54]. Moreover, the decrease of sepal length observed in salt-treated *S. lycopersicum* may limit sepal photosynthesis and reduce the supply of carbohydrates for fruit and seed growth as observed in hellebore [55]. It was indeed reported that photosynthesis of green reproductive organs contribute in a significant way to fruit growth [56,57]. A decrease of yield in *S. lycopersicum* subjected to salinity has frequently been described and was explained by a decrease in fruit size rather than by a decrease in fruit number [21,58], which corroborates our observations. Martínez et al. [21] compared fruit yield in *S. lycopersicum* and *S. chilense* in response to NaCl (0–80 mM) and observed that, although salt decreased fruit production and fruit weight in *S. lycopersicum*, it did not affect these parameters in *S. chilense*. *Solanum chilense* seems thus able to maintain its fruit production in salt conditions. Maintenance of fruit size and seed set under salt stress could be of great interest for tomato improvement. However, salinity affected fruit quality in both species. We observed that salinity increased fruit sugar concentrations in *S. lycopersicum* but decreased it in *S. chilense*; salt also decreased fruit pH in both species. Martínez et al. [21,26] also observed a change in fruit quality in both species as a response to salt. For example, they observed that both species differed regarding their main antioxidant compounds and that salinity increased the antioxidant capacity in *S. chilense* while it decreased it in *S. lycopersicum* [26].

### 3.2. Salinity Affects Mineral Accumulation and Distribution Which May Affect Fertility

The decrease of inflorescence and flower production and of flower fertility as well as the increase of flower abortion in response to abiotic stress is often explained in terms of competition for assimilates or alteration of carbohydrates metabolism [29,30,59]. However, in response to salinity, we may not exclude that the negative impact on flower production and fertility could be due to an accumulation of toxic ions in the reproductive structures [60,61].

The sodium concentration increased in the inflorescences and the fruits of salt treated plants of both species as soon as they were exposed to 60 mM NaCl, but final concentrations in *S. chilense* were higher than in *S. lycopersicum*. However, Na concentrations were lower in the seeds than in the pericarp, suggesting that the plant protect the next generation. A limitation of toxic ions in the seeds has indeed been reported in other plant species such as rice [62] and *Kosteletzkya pentacarpos* [63]. It was previously shown that *S. chilense* accumulated more Na in the vegetative aerial parts than *S. lycopersicum* during vegetative growth [64]. Our results showed that a similar situation occurred in the reproductive organs. The higher salinity resistance of *S. chilense* compared to *S. lycopersicum* regarding flower and fruit production can therefore not be explained by Na exclusion in the reproductive parts.

However, the Na distribution in the flowers differed in the species. In *S. lycopersicum*, Na was mostly accumulated in the non-reproductive parts of the flowers and especially in the pedicel and receptacle. This suggests that *S. lycopersicum* protects the reproductive organs by limiting Na accumulation in this sensitive tissue. Ghanem et al. [29] previously reported that *S. lycopersicum* limited Na accumulation in the reproductive organs and particularly in pollen grains. However, we may not exclude that the higher Na accumulation in the non-reproductive floral organs contributed to the decrease of sepal length. The sepal length was indeed negatively correlated with the Na signal ratio between floral receptacle and reproductive floral organs in *S. lycopersicum*. It is known that Na accumulation reduced vegetative growth in *S. lycopersicum* [64,65]. In *S. chilense*, Na accumulated more in reproductive floral organs and mainly in stamens. This could explain the decrease of stamen length and pollen production observed in salt-treated *S. chilense*. The number of pollen grains per stamen was indeed negatively correlated with the concentration of Na in the inflorescences in this species. In *S. lycopersicum*, the exclusion of Na in the male floral organs probably led to the protection of pollen because neither pollen viability nor the number of pollen grains per stamen were affected by salt stress in our study. It is often reported that male reproductive floral organs are more affected by abiotic stress than female floral organs in tomato [29,66], suggesting that the latter is more protected than the former. However, we observed that the ratio of the Na signals between male and female floral organs decreased with salt in both species. Regarding female floral organs, Na accumulated in the external tissues over the ovary but not in the ovules in *S. chilense*, whereas in *S. lycopersicum*, Na signal was low in female organs but was distributed in the whole ovary. Such differences in Na localization between species may explain the effects of salinity on fruit development in both species. Fruit and seed development were indeed more affected in *S. lycopersicum* than in *S. chilense*.

In addition to the accumulation of Na, modification of the concentration or localization of other key minerals may also affect flower development and fertility. Indeed, K is an essential macronutrient in flower development, particularly for stamen and pollen grains [67]. We observed that the concentrations of K and Na in inflorescences were negatively correlated and that the K/Na ratio decreased with salt stress in both species although K concentrations in inflorescences were not affected by salinity. In vegetative organs, a decrease of K is often observed in response to NaCl [2,29,64,68], which negatively affects C/N nutrition and the activity of several enzymes [69,70]. The maintenance of sufficient K concentration in inflorescences despite salt stress can be explained by the importance of this element for reproductive development and especially for elongation of filaments and release of pollen [67]. For example, K contributed to anther dehiscence and pollen imbibition in rice [71,72]. Decrease of the K/Na ratio is commonly reported as symptomatic of salinity stress [73]. Surprisingly, we observed that the K/Na ratio is more important in the inflorescences of *S. lycopersicum* than in those of *S. chilense*, even at high NaCl concentration. Albaladejo et al. [74] observed also a more significant decrease in K concentration with salinity in the halophyte *S. pennellii* than in *S. lycopersicum*. They hypothesized that this wild tomato species is able to withstand K deficiency by using Na in osmoregulation: K may indeed be replaced by Na in non-specific activities in a few species [69], notably in enzyme activities [75]. This could be a resistance strategy also shared by *S. chilense* to withstand the Na accumulation. Magnesium is also required for pollen development since mutants in the Mg transporter family genes, AtMGT, showed pollen-abortive phenotypes [76]. We observed that Mg accumulated in the stamens and the ovary of *S. chilense* and in the ovary of *S. lycopersicum*, suggesting also a potential role for ovary and fruit development. Because of its fundamental role in phloem export of carbohydrates, Mg is of critical importance during the reproductive growth stage of plants to maintain and maximize carbohydrates transport to sink organs [77]. Calcium is known to play a key role in pollination and pollen tube growth [78] as well as in fruit development [79]. We observed that Ca concentration and localization also differed between both species and Ca accumulated in ovaries in response to salt. Concentrations of Mg and Ca were higher in the inflorescences of *S. lycopersicum* than in the ones of *S. chilense*. However, more research is required to understand their role in flower and fruit development.

### 3.3. Mineral Transporters Are Involved in Na Accumulation and Partitioning in the Reproductive Structures

To better understand the localization of Na in tomato reproductive structures, we investigated the expression of genes coding for Na transporters. SOS1 is a Na^+^/H^+^ exchanger activated by the complex formed by SOS2 and SOS3 [80,81,82]. The SOS pathway is involved in Na exclusion out of the cell [83,84,85]. We observed that expression of *SOS1* and *SOS2* increased with salt stress in flowers of *S. lycopersicum* and to a lesser extent in the ones of *S. chilense*. Moreover, their expression was positively correlated with the Na concentration ratio between non-reproductive and reproductive floral organs in *S. lycopersicum*. Surprisingly, we found a decrease of *SOS3* expression with salt in *S. lycopersicum*, despite its role in activation of SOS1 [83]. However, pathways other than the SOS2–SOS3 complex are involved in Na^+^ activation of SOS1 [83]. Induction of the expression of *SOS1* and *SOS2* is commonly reported in response to salt stress in vegetative parts, and their overexpression induces a better salt resistance [81,86,87]. By contrast, knock-out mutants of these genes lead to a decrease in salt resistance [88,89]. Our results suggested that the SOS pathway is also activated in reproductive organs in response to salt stress. In contrast to our results, Romero-Aranda et al. [25] did not observe any induction of *SOS1* in inflorescences of tomato near-isogenic lines homozygous for *S. cheesmaniae SOS1* allele under salinity conditions. The involvement of *SOS1* in inflorescences thus seems species-dependent in the tomato clade and may differ among halophyte and glycophyte species. Based on those results, *SOS1* expression is induced in salt response in a higher extent in the glycophyte *S. lycopersicum* than in the halophytes *S. cheesmaniae* and *S. chilense* at the reproductive level. We indeed observed that the expression of *SOS* genes was correlated with Na concentrations in the inflorescences of *S. lycopersicum* but not of *S. chilense*.

Other genes involved in the Na transport at the cell level are *NHX3* and *NHX4*, which encode tonoplast transporters involved in the import of Na to the vacuole [90,91,92]. In our study, *NHX3* expression decreased and *NHX4* expression increased with salt stress in *S. lycopersicum* flowers while their expression was not affected by salinity in *S. chilense* flowers. This differs with the results of Gálvez et al. [91], who compared the response of *S. lycopersicum* and *S. pimpinellifolium* to salinity. They indeed observed that *NHX3* and *NHX4* were upregulated by salinity, especially in the wild halophyte *S. pimpinellifolium* [91]. However, they analyzed plants at the vegetative stage and did not investigate expression in the reproductive organs. We may thus not exclude that the involvement of *NHX* genes differ in vegetative and reproductive organs in tomato species subjected to salinity. Nevertheless, Bassil et al. [67] have shown that, in Arabidopsis, *AtNHX1* and *AtNHX2* are involved in flower development by regulating vacuolar pH and K^+^ homeostasis and that Na^+^ could partially substitute K^+^ in presence of salt. *AtNHX1* and *AtNHX2* are the closest *AtNHX* homologs of *SlNHX4* [91]. We could hypothesize that, in *S. lycopersicum*, under salt stress conditions, the increase of *NHX4* expression would be related to an attempt to increase the K concentration in the anthers, whereas the fact that *S. chilense* could use Na instead of K for flower development and therefore would not require high *NHX4* expression remains an open question.

Other transporters are involved in Na and K transport. HKT1;2 belongs to HKT1-like transporters whose role is to remove Na from the xylem in the roots [93]. However, it has been shown that this gene family is important in salinity resistance during the reproductive stage [25,94]. In our study, *HKT1;2* expression decreased with salinity in both species, possibly explaining the accumulation of Na in the inflorescences. This gene seems to be involved in the partitioning of Na in the flowers as its expression was positively correlated with the Na ratio between male and female floral organs and negatively correlated with the Na ratio between vegetative and reproductive floral organs in both species. *SlAKT2/3* is a phloem K transporter involved in long-distance transport of sucrose [40]. This gene is expressed in tomato flowers and especially sepals [95,96]. We observed that the expression of *SlAKT2/3* increased with NaCl in *S. lycopersicum* but not in *S. chilense*. In the same way, the expression of *SlHAK14* increased with salt stress in *S. lycopersicum* only. SlHAK14 and SlHAK3 are K transporters belonging to the KT/KUP/HAK family [97], and they are both very highly expressed in pollen [96]. In *S. chilense*, the expression of both *SlHAK14* and *SlHAK3* decreased at a concentration of 60 mM NaCl compared to the other treatments. The expression of *SlHAK14* negatively correlated with the K concentration in inflorescences in *S. lycopersicum* only, suggesting a different role in element regulation in *S. lycopersicum* and in *S. chilense*. The expression of *CNGC10* also differed among tomato species. It increased with salinity in *S. chilense* but not in *S. lycopersicum*. This gene is linked to the import of Na and K in flowers, and its expression is inhibited by salinity in Arabidopsis [98]. Its higher expression in *S. chilense* could partly explain the higher Na concentration in inflorescences and flowers of *S. chilense* compared to *S. lycopersicum*. Our results suggest that Na and K transport could be differently regulated in flowers of *S. lycopersicum* and *S. chilense*. Moreover, correlations between transporters expression and mineral concentrations in flowers differed in both species, mainly for the SOS pathway. Further studies are required to decipher the role of transporters in Na and K localization in flowers of both species.

## 4. Materials and Methods

### 4.1. Plant Material and Growth Conditions

Seeds of *Solanum lycopersicum* L. cv Ailsa Craig (accession LA2838A) and of *Solanum chilense* Dunal (accession LA4107) were obtained from the Tomato Genetics Resource Center (TGRC, University of California, Davis, CA, USA) and INIA-La Cruz (La Cruz, Chile), respectively. *S. chilense* was subjected to 6 days pre-germination in Petri dishes on humid filter paper at 25 °C and 12 h photoperiod before sowing in peat compost (DCM, Amsterdam, The Netherlands) and transferred to a temperate greenhouse. Sowing of *S. lycopersicum* was performed in the same peat compost and in the same greenhouse 13 days after the sowing of *S. chilense* so they would be of the same developmental stage at the start of stress application. When the two-leaf stage was reached, the plants were individually transplanted in pots (2.5 L) on perlite/vermiculite (50% *v*/*v*) and were grown under the same temperate greenhouse conditions (24 ± 1.5 °C, 63 ± 8% RH day, 21 ± 0.8 °C, 67 ± 5% RH night, 16 h-photoperiod). In addition to natural light, supplementary lighting was provided by LED LumiGrow lights (650 W, red-blue) to maintain a minimum light intensity (mean light in the middle of a cloudy day 181.33 ± 63.42 µmol m^−2^ s^−1^). Plants were watered three times a week with modified Hoagland solution (5 mM KNO_3_, 5.5 mM Ca(NO_3_)_2_, 1 mM NH_4_H_2_PO_4_, 0.5 mM MgSO_4_, 25 µM KCl, 10 µM H_3_BO_4_, 1 µM MnSO_4_, 0.25 µM CuSO4, 1 µM ZnSO_4_, 10 µM (NH_4_)_6_Mo_7_O and 1.87 g L^−1^ Fe-EDTA, and pH 5.5–6). After four days of acclimation, plants were randomly divided into four groups (25 plants per group) receiving 0, 60, 100, or 120 mM NaCl (respectively, 0.86, 7.07, 10.82, and 12.72 mS cm^−1^). Salt solutions were applied three times a week at the same time that the Hoagland solution, with volumes depending on the physiological stage of the plant.

### 4.2. Growth

Vegetative growth was assessed by counting the number of leaves on the main stem on 10 plants per condition and species, once a week. Reproductive growth was also assessed on the same 10 plants per condition and species. Flowering time of the initial and the sympodial segments were assessed by counting the number of leaves below the first inflorescence and between inflorescences, respectively. The number of inflorescences on the main stem was counted once a week from 20 days after stress imposition (DASt). The number of flower buds and flowers at anthesis per inflorescence was followed on the second and third inflorescences.

Per condition and species, 11 to 20 flowers at anthesis from the second inflorescence of the main stem were harvested to evaluate the length of sepals, petals, stamens, pistil, and ovary. The style exertion was also assessed for *S. chilense* by measuring the length of the pistil outside the stamen cone. Organs were dissected, flattened, and measured using ImageJ (version 1.53a).

### 4.3. Flower Fertility

To detect stigma receptivity, peroxidase activity was tested at the stigma’s surface according to Dafni and Maués [99]. At anthesis, 14 to 22 flowers per condition were harvested. Stigmas were dissected and immersed for 5 min in acetate buffer with 112.2 mM CaCl_2_·2H_2_O, 2.3 mM 3-amino-9-ethylcarbazole diluted in N-N-dimethylformamide, and 0.014% H_2_O_2_ (*v*/*v*). The reddish-brown color developed on the surface was scored by 0 (no receptive stigma) or 1 (receptive stigma). Pollen viability was assessed on two stamens of the same flowers using Alexander dye [100]. Pollen was considered viable when a red coloration appeared, whereas it was considered non-viable when its coloration was green. A minimum of 100 pollen grains was counted by anther. The number of pollen grains per anther was determined by crushing an anther in 40 µL of Alexander’s dye and counting using ImageJ as described by Ayenan et al. [101], showing a pollen size of 5–800 pixel^2^ and a circularity of 0.3–1.0. Six pictures were taken by anther, and two anthers per flower and 10 flowers per condition and species were analyzed.

### 4.4. Fruit Parameters

For fruit production, flowers of *S. lycopersicum* were self-pollinated, and flowers of the self-incompatible *S. chilense* were hand pollinated with pollen from the same condition. The fruit set was assessed by the ratio between the number of obtained fruits and the number of pollinated flowers. Fruits were collected at the maturity stage. The number of seeds per fruit, circumference, and fresh weight (FW) were measured for 10 to 15 fruits per condition and species. For the same fruits, sugar concentration was estimated in degrees Brix by refractometry (Eclipse, Bellingham + Stanley, Tunbridge Wells, UK), and the pH of the juice was evaluated by pH paper (Dosatest pH test strips pH 3.6–6.1, VWR).

### 4.5. Mineral Elements Concentrations and Element Distribution

Sodium (Na), potassium (K), calcium (Ca), and magnesium (Mg) were quantified in inflorescences, pericarp, and seeds of fruits. Material was oven-dried at 70 °C for 72 h, and 50 to 100 mg dry weight (DW) was weighted and digested in 4 mL of warm 68% (*v*/*v*) HNO_3_. After complete dissolution, minerals were dissolved in aqua regia (HCl 37%:HNO_3_ 68% 3:1), filtered (Whatman, 11 µm), and quantified by flame atomic absorption spectrophotometry (ICE 3300, Thermo Scientific, Waltham, MA, USA) using suitable standards (Spectracer-CPACHEM; accredited through ISO/IEC17025). Quantification was performed on at least nine samples per condition and species.

Flowers of both species growing at 0, 60, and 100 mM were longitudinally cut using a platinum coated razor blade and sandwiched between two aluminum foils, flattened, frozen in liquid nitrogen, and freeze-dried (−30 °C, 0.210 mbar, Alpha 2–4, Christ, Osterode am Harz, Germany) for 72 h. Two flowers per condition and species were placed on double sided Scotch^®^ tape on glass slides, and the distribution of Na, Mg, K, and Ca was evaluated by laser ablation inductively coupled plasma mass spectrometry (LA-ICP-MS, Agilent 7900×, Agilent Technologies, Palo Alto, CA and Analyte G2, Teledyne Photon Machines Inc., Bozeman, MT, USA). The laser ablation system contains a HelEx II 2-volume ablation cell with integrated Aerosol Rapid Introduction System [102]. The imaging parameters for best image quality were set according to van Elteren et al. [103] (LA settings: square 20 μm beam size, 275 Hz, dosage 11, 1 J/cm^2^; ICP-MS: acquisition time 40 ms, dwell times Mg, Na, K, 7 ms and Ca 12 ms). Distribution of elements was visualized using ImageJ [104] by adjusting contrasts and using Look Up Table (LUT) menu. Colocalisation maps (Na with K, Mg, or Ca) were generated by merging channels in ImageJ. Number of counts in specific organs was estimated in two flowers per condition and per species using ROI (Regio Of Interest) manager by selecting an ovary, a style, one stamen, and a floral receptacle. The ratio between the number of counts of each element in the male part (one stamen) and female parts (ovary and style) and the ratio between vegetative (floral receptacle) and reproductive (stamen, ovary, and style) parts were determined.

### 4.6. Transporters Expression Analysis by qRT-PCR

The expression of 10 genes coding for mineral transporters was analyzed. Genes were selected according to the literature [25,37,40,98,105,106,107] and on transcriptome profiling of inflorescences of tomato during salt stress imposition [96]. When the sequences were not described in tomato, sequences of tomato homologs were identified using nucleotide BLAST again National Center for Biotechnology Information (NCBI) and Sol Genomics Network (SGN) databases and alignment with BioEdit. A first bioinformatics study of the expression of these genes was analyzed via available databases (TomExpress, SGN, [96]). The obtained full-length tomato sequences were used for primer design using Primer3Plus [108]. The analyzed genes and primer sequences are described in Table S1.

Flowers at anthesis were collected at 35 DASt and stored in liquid nitrogen. RNA extraction was performed on three samples of 100 mg of flowers per condition and species using TRI Reagent Solution (Ambion, Austin, TX, USA) with DNase treatment (RQ1 DNase 1 U/µg Promega, Leiden, The Netherlands) according to the manufacturer’s instructions. First-strand cDNA was synthesized from 1 µg RNA using the Revertaid H Minus First Strand cDNA Synthesis Kit (ThermoFisher, Waltham, MA, USA). The concentration and purity of the RNA were measured using a NanoDrop ND-1000 spectrophotometer (Thermo Scientific, Villebon-sur-Yvette, France). Transcript levels were quantified in two independent qPCR (in triplicates for each of the three biological replicates) using the GoTaq qPCR Master Mix (Promega) in StepOnePlus Real-Time PCR systems (Applied Biosystems, Foster City, CA, USA). Cycling conditions were initial denaturation 10 min at 95 °C, then 40 cycles of 15 s at 95 °C, and 1 s at 60 °C. The tomato housekeeping genes *LeEF1-α* (Elongation factor 1-alpha, Solyc06g005060) and *TIP41* (TIP41-like protein, Solyc10g049850) were used as reference genes [109]. Results were expressed using the ΔΔCt calculation method in arbitrary units by comparison to the expression of *S. lycopersicum* under control conditions, and normalization was carried out with *LeEF1-α* and *TIP41*. A melt-curve analysis was performed to check the specific amplifications.

### 4.7. Statistical Analysis

All statistical analyses were performed in RStudio (R Development Core Team, 2017). Normality distribution and homoscedasticity were verified using the Shapiro–Wilk and Levene’s tests, respectively, and data were transformed when required. When possible, two-way analysis of variance (ANOVA II) was used to compare species, salinity, and their interactions. Comparisons between the two species were analyzed using the Student’s test, the permutation Student’s *t*-test (if normality was not met), or the Wilcoxon test (if homoscedasticity was not met). For a single species, comparisons between NaCl treatments were made using one-way analysis of variance (ANOVA I), ANOVA I using the permutation test (if normality was not met), or the Kruskal–Wallis test (if homoscedasticity was not met), followed by appropriate post-hoc tests. Data are shown as means ± standard deviation. For results obtained by LA-ICP-MS, no statistical treatment was applied because of the lack of repetitions (two repetitions per condition and species). Statistical results are presented in Table S2.

## Figures and Tables

**Table 1:** Effects of salt stress on flowering parameters of *Solanum lycopersicum* and *Solanum chilense* grown at 0, 60, and 120 mM NaCl.

Flowering Parameters	*S. lycopersicum*			*S. chilense*		
	0 mM NaCl	60 mM NaCl	120 mM NaCl	0 mM NaCl	60 mM NaCl	120 mM NaCl
FT initial segment ^1^	11.2 ± 1.3 ^a^	10.8 ± 1.2 ^a^	11.0 ± 0.9 ^a^	12 ± 1.1 ^A^	10.0 ± 1.5 ^A^	11.8 ± 1.7 ^A^
FT sympodial segment ^1^	2.7 ± 0.3 ^a^	3.0 ± 0.3 ^a^	3.0 ± 0.4 ^a^	3.8 ± 1.2 ^A^	3.1 ± 0.5 ^A^	4.2 ± 2.4 ^A^
inflorescences per plant	96.0 ± 61.9 ^a^	28.8 ± 5.3 ^a^	10.0 ± 1.4 ^b^	160.3 ± 58.9 ^A^	53.5 ± 17.9 ^A^	7.8 ± 4.9 ^B^
floral buds per inflorescence	8.32 ± 2.29 ^a^	6.56 ± 1.19 ^b^	6.14 ± 1.39 ^b^	12.5 ± 7.18 ^A^	11.38 ± 9.40 ^A^	10.40 ± 5.58 ^A^
open flowers per inflorescence (%)	74.6 ± 19.4 ^a^	55.6 ± 25.5 ^b^	50.7 ± 27.1 ^b^	71.3 ± 29.6 ^A^	54.5 ± 34.0 ^A^	47.5 ± 30.8 ^A^

^1^ FT: flowering time; expressed in number of leaves; data are means ± standard deviation, different letters indicate significant difference for each species (lowercase, *S. lycopersicum*, uppercase, *S. chilense*) at *p* < 0.05.

**Table 2:** Effects of salt stress on flowering morphology and fertility of *Solanum lycopersicum* and *Solanum chilense* grown at 0, 60, and 120 mM NaCl.

Flower Parameters	*S. lycopersicum*			*S. chilense*		
	0 mM NaCl	60 mM NaCl	120 mM NaCl	0 mM NaCl	60 mM NaCl	120 mM NaCl
Sepal length (cm)	1.18 ± 0.23 ^a^	0.89 ± 0.16 ^b^	0.91 ± 0.15 ^b^	0.62 ± 0.1 ^A^	0.61 ± 0.08 ^A^	0.63 ± 0.15 ^A^
Petal length (cm)	1.36 ± 0.18 ^a^	1.24 ± 0.23 ^a^	1.34 ± 0.17 ^a^	1.12 ± 0.2 ^A^	1.25 ± 0.22 ^A^	1.18 ± 0.17 ^A^
Stamen length (cm)	0.84 ± 0.09 ^a^	0.8 ± 0.08 ^a^	0.85 ± 0.07 ^a^	0.80 ± 0.05 ^AB^	0.82 ± 0.08 ^A^	0.74 ± 0.06 ^B^
Style + ovary length (cm)	0.94 ± 0.07 ^a^	0.86 ± 0.1 ^a^	0.94 ± 0.09 ^a^	1.18 ± 0.11 ^A^	1.11 ± 0.12 ^A^	1.08 ± 0.09 ^A^
Style exsertion (cm)	ND	ND	ND	0.38 ± 0.12 ^A^	0.29 ± 0.15 ^A^	0.33 ± 0.09 ^A^
Stigma receptivity (%)	88.6 ± 26.4 ^a^	81 ± 29.5 ^a^	84.4 ± 30.1 ^a^	96.4 ± 13.4 ^A^	100 ± 0 ^A^	100 ± 0 ^A^
Pollen viability (%)	84.7 ± 13.5 ^a^	82.5 ± 21.2 ^a^	81.6 ± 14.2 ^a^	58.3 ± 26.1 ^B^	68.9 ± 25 ^A^	63 ± 14.3 ^AB^
Pollen grains per anther (×1000)	19.2 ± 14.2 ^a^	13.9 ± 15.2 ^a^	16.3 ± 10.2 ^a^	68.0 ± 35.1 ^A^	48.5 ± 23.8 ^AB^	37.4 ± 19.3 ^B^

ND, no style exsertion. Data are means ± standard deviation, different letters indicate significant difference for each species (lowercase, *S. lycopersicum*, uppercase, *S. chilense*) at *p* < 0.05.

**Table 3:** Effects of salt stress on fructification parameters of *Solanum lycopersicum* and *Solanum chilense* grown at 0, 60, and 120 mM NaCl.

Fruit Parameters	*S. lycopersicum*			*S. chilense*		
	0 mM NaCl	60 mM NaCl	120 mM NaCl	0 mM NaCl	60 mM NaCl	120 mM NaCl
Fruit set (%)	47.9 ± 15.1 ^a^	43.5 ± 22.5 ^a^	38.9 ± 21.1 ^a^	51.7 ± 40.6 ^A^	60 ± 37.7 ^A^	44.3 ± 33.2 ^A^
FW (g)	47.7 ± 10.3 ^a^	22.5 ± 6 ^b^	14.5 ± 5.3 ^c^	0.65 ± 0.2 ^A^	0.79 ± 0.26 ^A^	0.84 ± 0.21 ^A^
DW (g)	3.42 ± 1.98 ^a^	2.01 ± 0.56 ^b^	1.44 ± 0.62 ^b^	0.12 ± 0.03 ^A^	0.10 ± 0.02 ^A^	0.10 ± 0.01 ^A^
WC (%)	91.91 ± 3.77 ^a^	89.67 ± 0.42 ^b^	88.19 ± 0.9 ^c^	80.72 ± 2.87 ^C^	82.49 ± 7.17 ^B^	87.49 ± 1.6 ^A^
Circumference (cm)	14.4 ± 0.92 ^a^	11.62 ± 0.74 ^b^	10.1 ± 1.13 ^c^	3.15 ± 0.24 ^A^	3.48 ± 0.73 ^A^	3.57 ± 0.62 ^A^
Number of seeds/fruit	91.17 ± 46.02 ^a^	72.77 ± 33.19 ^ab^	50.08 ± 16.04 ^b^	21.22 ± 4.47 ^A^	22.00 ± 5.28 ^A^	24.88 ± 10.21 ^A^
Number of seeds/fruit FW (g)	2.11 ± 0.73 ^b^	3.31 ± 0.91 ^a^	3.76 ± 2.1 ^a^	34.37 ± 13.44 ^A^	30.72 ± 11.83 ^A^	25.93 ± 7.18 ^A^
Sugar concentration (°Brix)	5.54 ± 0.52 ^c^	7.95 ± 0.44 ^b^	9.2 ± 0.95 ^a^	18.4 ± 3.2 ^A^	11.95 ± 4.12 ^B^	10.15 ± 2.35 ^B^
pH	4.52 ± 0.09 ^a^	4.4 ± 0.11 ^b^	4.31 ± 0.11 ^b^	4.67 ± 0.26 ^A^	4.07 ± 0.49 ^B^	3.8 ± 0.26 ^B^

Data are means ± standard deviation; different letters indicate significant difference for each species (lowercase, *S. lycopersicum*, uppercase, *S. chilense*) at *p* < 0.05. DW, FW, dry and fresh weights; WC, water content.

**Table 4:** Effects of salt stress on ratio (vegetative/reproductive organs and male/female organs) of mineral elements signals in flowers of *Solanum lycopersicum* and *Solanum chilense* grown at 0, 60, and 100 mM NaCl.

Mineral	*S. lycopersicum*			*S. chilense*		
	0 mM NaCl	60 mM NaCl	100 mM NaCl	0 mM NaCl	60 mM NaCl	100 mM NaCl
vegetative/reproductive floral organs						
Na	0.36 ± 0.12	0.65 ± 0.11	1 ± 0.26	0.18 ± 0.07	0.43 ± 0.04	0.31 ± 0.02
K	0.45 ± 0.1	0.43 ± 0.05	0.5 ± 0.03	0.43 ± 0.19	0.22 ± 0.09	0.14 ± 0.02
Ca	0.54 ± 0.07	0.42 ± 0.05	0.69 ± 0.24	0.34 ± 0.29	0.29 ± 0.12	0.26 ± 0.08
Mg	0.86 ± 0.16	0.34 ± 0.1	0.77 ± 0.19	0.67 ± 0.46	0.23 ± 0.02	0.3 ± 0.08
male/female floral organs						
Na	0.82 ± 0.05	0.55 ± 0.16	0.5 ± 0.07	1.24 ± 0.68	0.59 ± 0.27	0.7 ± 0.49
K	0.63 ± 0.03	0.41 ± 0	0.34 ± 0.2	0.83 ± 0.06	0.6 ± 0.16	0.68 ± 0.54
Ca	0.64 ± 0.24	0.26 ± 0.02	0.3 ± 0.14	1.32 ± 0.07	0.35 ± 0.11	0.68 ± 0.29
Mg	0.49 ± 0.05	0.35 ± 0.12	0.3 ± 0.09	0.62 ± 0.2	0.38 ± 0.04	0.52 ± 0.37

Relative signal intensities obtained by LA-ICP-MS (laser ablation inductively coupled plasma mass spectroscopy) are expressed in counts. Signal intensities are correlated with the concentrations of a particular element (comparisons could be performed per element but not between elements).

**Table 5:** Effects of salt stress K, Ca, and Mg concentrations of different organs of *Solanum lycopersicum* and *Solanum chilense* grown at 0, 60, and 120 mM NaCl.

Mineral	*S. lycopersicum*			*S. chilense*		
	0 mM NaCl	60 mM NaCl	120 mM NaCl	0 mM NaCl	60 mM NaCl	120 mM NaCl
Inflorescences						
K (mg g^−1^ DW)	27.63 ± 2.62 ^a^	26.09 ± 6 ^a^	23.97 ± 4.36 ^a^	30.76 ± 5.06 ^A^	31.97 ± 4.81 ^A^	26.47 ± 5.21 ^A^
K/Na	43.01 ± 23.32 ^a^	10.34 ± 2.87 ^b^	10.15 ± 5.7 ^b^	10.66 ± 17.26 ^A^	4.74 ± 4.43 ^B^	1.26 ± 1.14 ^B^
Ca (mg g^−1^ DW)	1.03 ± 0.87 ^a^	1.22 ± 0.82 ^a^	1.09 ± 1.02 ^a^	0.18 ± 0.14 ^A^	0.08 ± 0.08 ^A^	0.29 ± 0.01 ^A^
Mg (mg g^−1^ DW)	4.92 ± 1.22 ^a^	5.62 ± 1.96 ^a^	3.64 ± 0.62 ^b^	3.45 ± 1.04 ^A^	2.94 ± 0.42 ^A^	3.04 ± 0.94 ^A^
Pericarp						
K (mg g^−1^ DW)	38.98 ± 6.36 ^a^	32.14 ± 9.24 ^b^	26.20 ± 5.88 ^b^	39.22 ± 4.66 ^A^	27.75 ± 4.47 ^B^	27.99 ± 6.74 ^B^
Ca (mg g^−1^ DW)	0.73 ± 0.23 ^a^	0.49 ± 0.23 ^b^	0.64 ± 0.24 ^ab^	1.49 ± 0.56 ^A^	1.27 ± 0.23 ^A^	1.54 ± 0.56 ^A^
Mg (mg g^−1^ DW)	1.36 ± 0.26 ^a^	1.09 ± 0.43 ^b^	1.08 ± 0.12 ^b^	2.10 ± 0.33 ^A^	2.14 ± 0.58 ^A^	2.46 ± 0.40 ^A^
seeds						
K (mg g^−1^ DW)	8.09 ± 5.09 ^a^	9.79 ± 7.28 ^a^	14.42 ± 9.2 ^a^	20.43 ± 7.6 ^A^	9.85 ± 5.68 ^B^	8.71 ± 7.21 ^B^
Ca (mg g^−1^ DW)	0.77 ± 0.53 ^a^	0.71 ± 0.54 ^a^	0.52 ± 0.13 ^a^	0.89 ± 0.23 ^A^	0.85 ± 0.24 ^A^	0.85 ± 0.39 ^A^
Mg (mg g^−1^ DW)	3.60 ± 0.93 ^a^	3.57 ± 0.92 ^a^	2.40 ± 0.75 ^a^	2.74 ± 0.27 ^A^	2.38 ± 0.26 ^B^	2.69 ± 0.37 ^AB^

Concentrations (mg g^−1^ DW) are measured by AAS (atomic absorption spectrometry). Data are means ± standard deviation, different letters indicate significant difference for each species (lowercase, *S. lycopersicum*, uppercase, *S. chilense*) at *p* < 0.05. DW, dry weight.

## Data Availability

The datasets generated during and/or analyzed during the current study are available from the corresponding author on reasonable request.

## Supplementary Materials

The following supporting information can be downloaded at: https://www.mdpi.com/article/10.3390/plants11050672/s1, Figure S1: Distribution of sodium (Na) in flowers of Solanum lycopersicum (top row) and Solanum chilense (bottom row) grown in perlite:vermiculite mixture supplied with 0, 60 and 100 mM NaCl as revealed by LA-ICP-MS (Laser ablation inductively coupled plasma mass spectroscopy) and visualized using ImageJ (version 1.53a) by using the same scale for all treatments. Colour legend represents the number of counts per pixel (20 × 20 µm^2^), the number of counts is linearly proportional to the Na concentration. Flowers are the same than in Figure 3, Figure S2: Distribution of potassium (K) in flowers of Solanum lycopersicum (top row) and Solanum chilense (bottom row). For details, see the legend of Figure S1, Figure S3: Distribution of calcium (Ca) in flowers of Solanum lycopersicum (top row) and Solanum chilense (bottom row). For details, see the legend of Figure S1, Figure S4: Distribution of magnesium (Mg) in flowers of Solanum lycopersicum (top row) and Solanum chilense (bottom row). For details, see the legend of Figure S1, Table S1: List of genes and their primers used for qRT-PCR and their efficiency, Table S2: Statistical results for the analyzed parameters.
