# Supplementary material for: The Halophyte Species Solanum chilense Dun. Maintains Its Reproduction despite Sodium Accumulation in Its Floral Organs
Source: Plants (Basel). 2022 Feb 28;11(5):672. doi: 10.3390/plants11050672 (PMC8912488; doi:10.3390/plants11050672)
Supplement: Supplementary file 1 [file plants-11-00672-s001.zip › plants-1608835-SI.pdf]

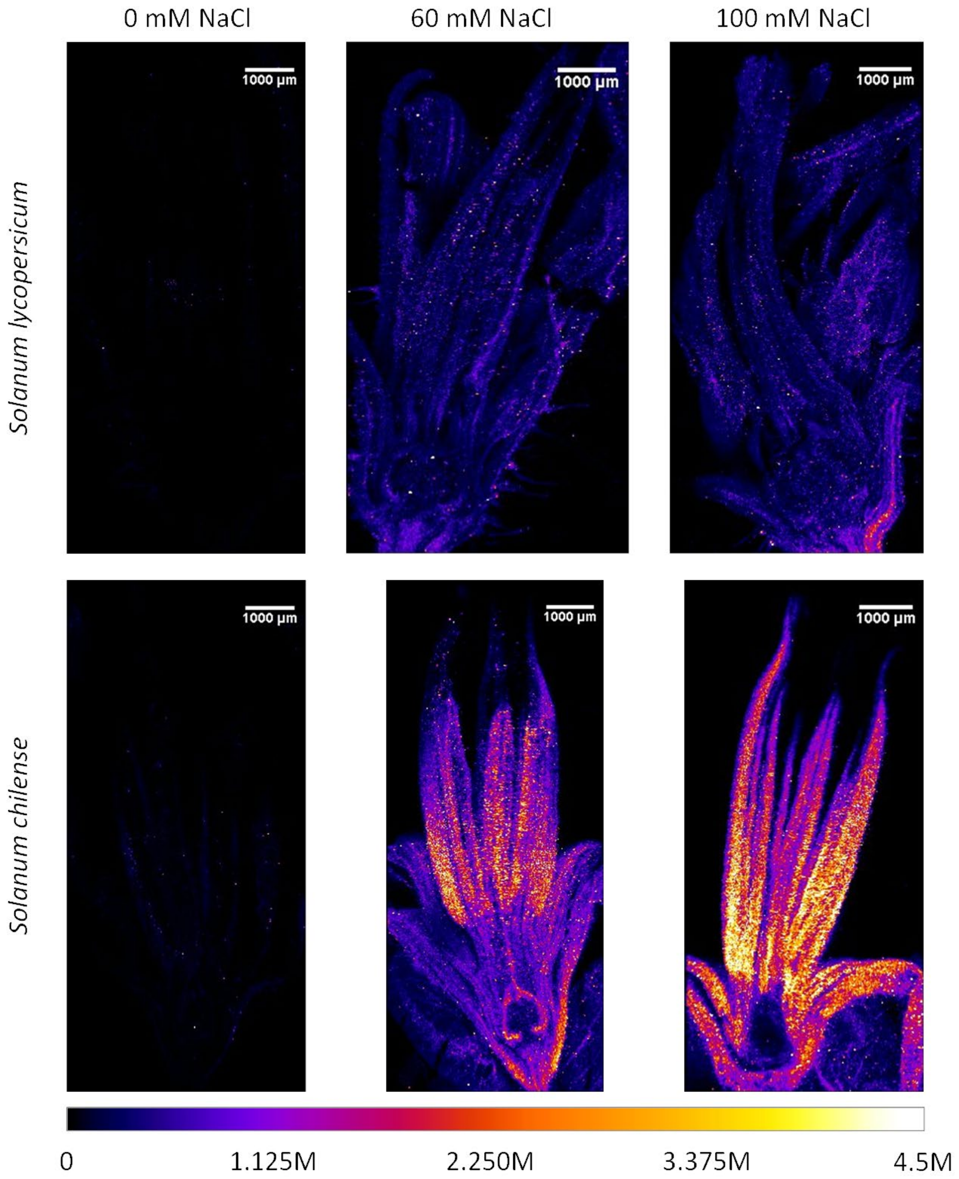

Figure S1. Distribution of sodium (Na) in flowers of *Solanum lycopersicum* (top row) and *Solanum chilense* (bottom row) grown in perlite:vermiculite mixture supplied with 0, 60 and 100 mM NaCl as revealed by LA-ICP-MS (Laser ablation inductively coupled plasma mass spectroscopy) and visualized using ImageJ (version 1.53a) by using the same scale for all treatments. Colour legend represents the number of counts per pixel (20x20 µm), the number of counts is linearly proportional to the Na concentration. Flowers are the same than in Figure 3.

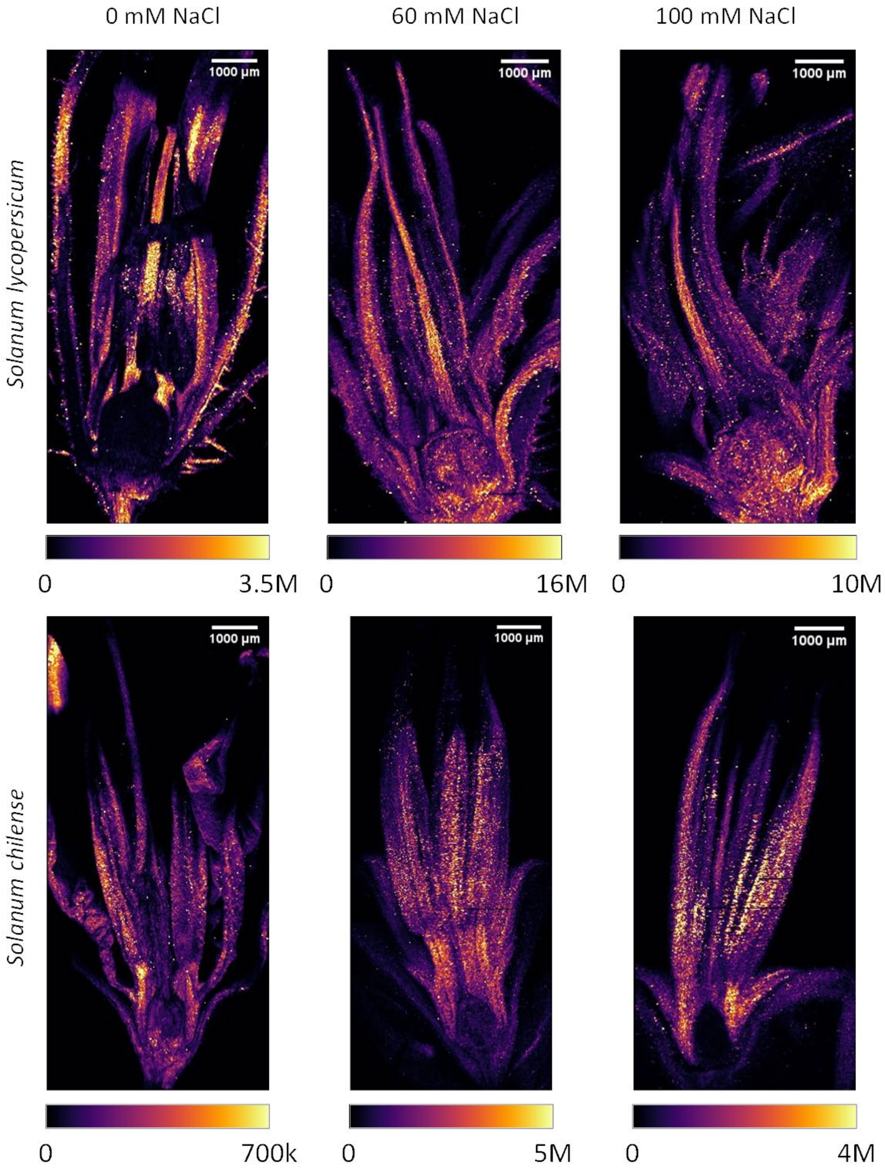

Figure S2. Distribution of potassium (K) in flowers of *Solanum lycopersicum* (top row) and *Solanum chilense* (bottom row) grown in perlite:vermiculite mixture supplied with 0, 60 and 100 mM NaCl as revealed by LA-ICP-MS (Laser ablation inductively coupled plasma mass spectroscopy) and visualized using ImageJ (version 1.53a). For details, see the legend of Figure S1.

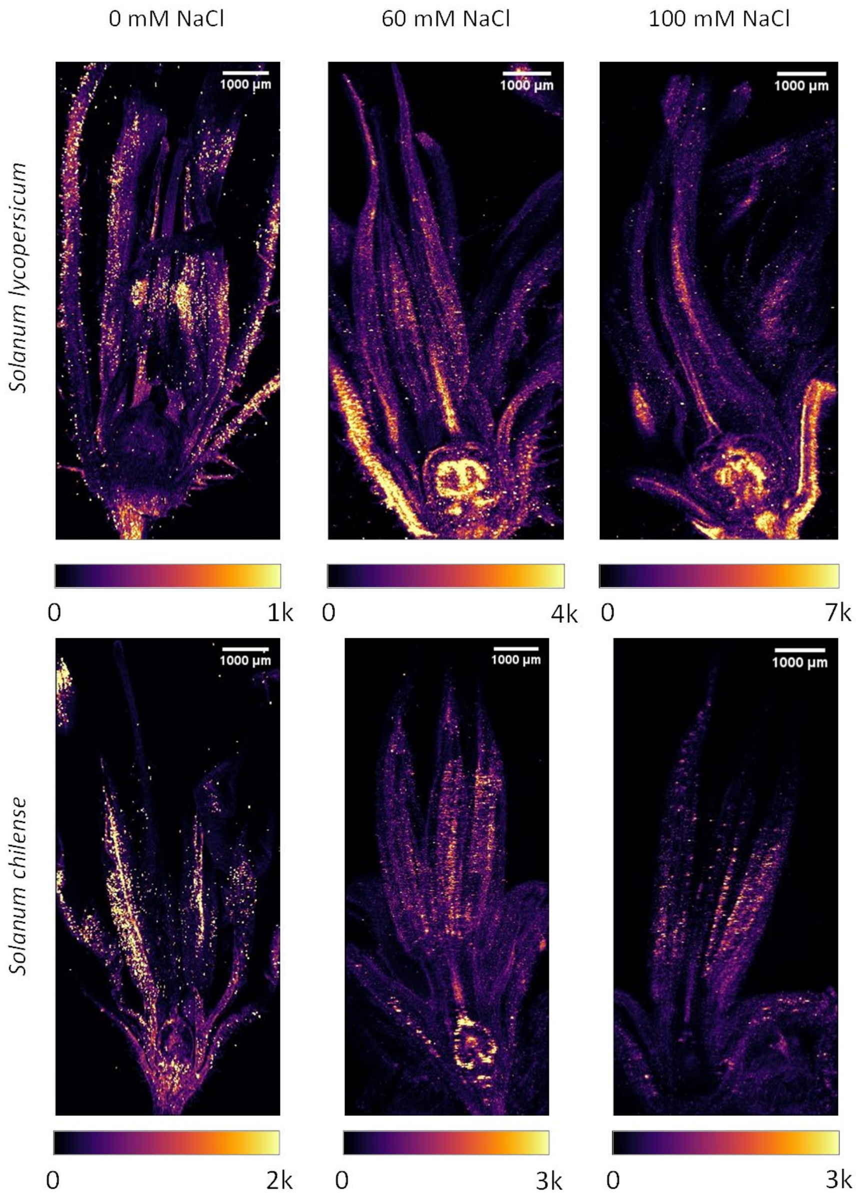

Figure S3. Distribution of calcium (Ca) in flowers of *Solanum lycopersicum* (top row) and *Solanum chilense* (bottom row) grown in perlite:vermiculite mixture supplied with 0, 60 and 100 mM NaCl as revealed by LA-ICP-MS (Laser ablation inductively coupled plasma mass spectroscopy) and visualized using ImageJ (version 1.53a). For details, see the legend of Figure S1.

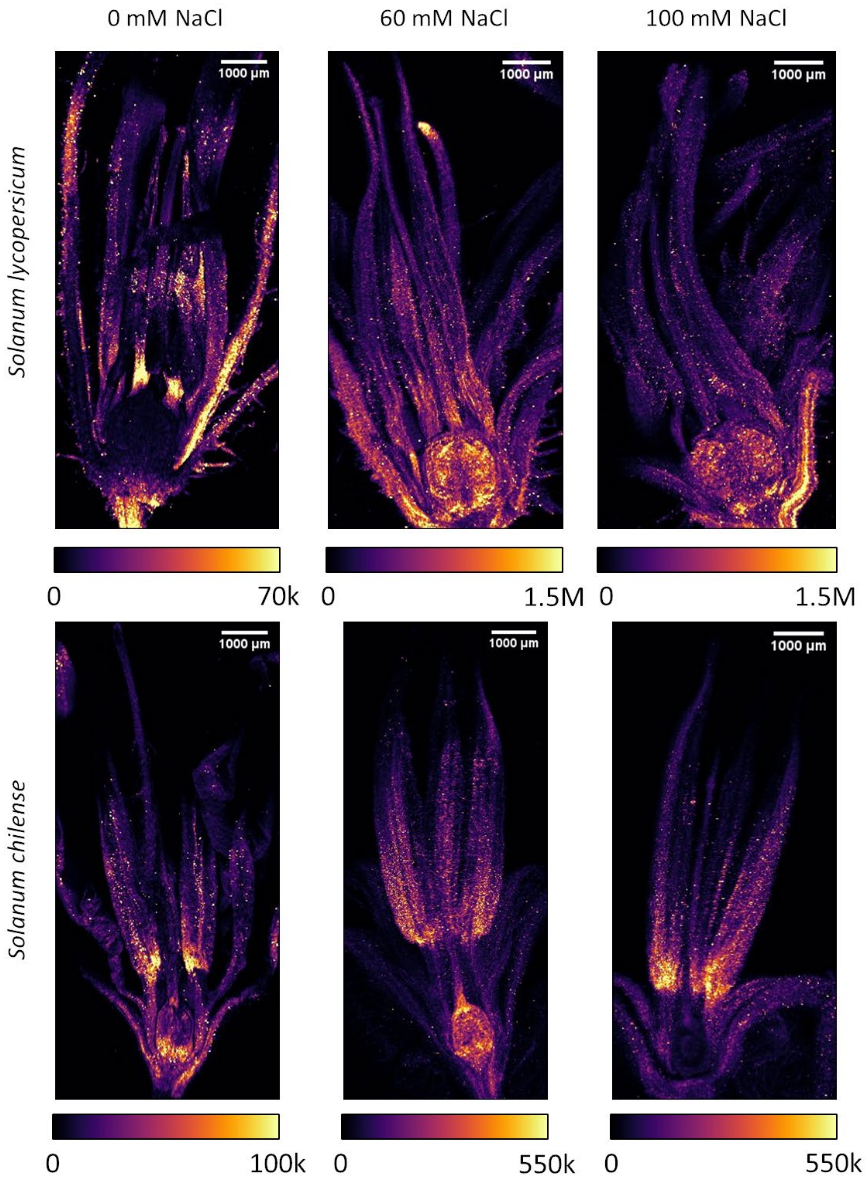

Figure S4. Distribution of magnesium (Mg) in flowers of *Solanum lycopersicum* (top row) and *Solanum chilense* (bottom row) grown in perlite:vermiculite mixture supplied with 0, 60 and 100 mM NaCl as revealed by LA-ICP-MS (Laser ablation inductively coupled plasma mass spectroscopy) and visualized using ImageJ (version 1.53a). For details, see the legend of Figure S1.

Table S1. List of genes and their primers used for qRT-PCR and their efficiency.

Name gene	Accession Number	primers	Amplicon length (bp)	Gene description	Reference
SOS1	Solyc01g005020	F: TCGAGTGATGATTCTGGTGG<br>R: GAGCCTTTCCACACTGTGAT	129	Na^+/H^+ exchanger in the plasmalemma	[25]
SOS2	Solyc12g009570	F: CTGCTTAGGACAAGGACTCG<br>R: GCAGTTACACACACTATACC	112	Serine/threonine kinase	[49]
SOS3	Solyc06g051970	F: ACACACCCGGATATGAGGAG<br>R: TTCCCCGAACTCAATCACTC	242	Calcium sensor	[49]
HKT1;2	Solyc07g014680	F: TGAGCTAGGGAATGTAATAAACG<br>R: AGAGAGAAACTAACGATGAACC	188	Na^+ transporter	[25,51]
NHX3	Solyc01g067710	F: TATGCGAGGTGCTGTTTCAG<br>R: AGTGTTTTGGTGAGGGTTGC	182	Tonoplast Na^+/H^+ exchanger	[104]
NHX4	Solyc01g098190	F: TGGTGGGCAGGTTTGATGAGAG<br>R: TGTGGTGGCAGCAGGAGACTTA	188	Tonoplast Na^+/H^+ exchanger	[25,100,104]
SlHAK14	Solyc09g074820	F: GTCACATACCCGGCTCTCAT<br>R: TGGCTGCGAATACAGCTATG	145	K^+ transport in membranes ; expression in pollen	[109]
SlHAK3	Solyc12g096580	F: CCATTCAAAGCAGCAAGTCA<br>R: TCCTTTGCCTGAACCAAATC	163	K^+ transport in membranes ; expression in pollen	[109]
SlAKT2/3	Solyc10g024360	F: GCAATGCCACTTTCCTTGAT<br>R: ATGCGGTGTCACCATTTACA	177	K^+ transport ; activation in *S. chilense* subjected to salinity	[40]
CNGC10	Solyc05g050350	F: GCTGGTGACTTCTGTGGTGA<br>R: TGTGAGGCGACAAACTTGAG	155	Negative regulation of salt tolerance in *Arabidopsis thaliana*	[50]
TIP41	Solyc10g049850	F: CGAAAGCAATGTCGAGTGTG<br>R: GGTTCTTTAGACGCCAATGC	111	TIP41-like protein	[54]
LeEF1-α	Solyc06g005060	F: GACAGGCGTTCAGGTAAGGA<br>R: GGGTATTCAGCAAAGGTCTC	119	Elongation factor 1-α	[49]

Table S2. Statistical results for the analyzed parameters.

Parameter	Genotype	NaCl	genotype x NaCl	Analysis
**Vegetative and reproductive growth**				
Number of leaves 113 DASt	F = 10.885, **	F = 15.997, ***	F = 0.769, p=0.473	Anova 2
# inflorescences 113 DASt	F = 73.540, ***	F = 17.624, ***	F = 2.727, P = 0.082	Anova 2
# leaves total 85 DASt	F = 6.894, *	F = 10.818, **	F = 0.951, p = 0.413	Anova 2
**Flowering parameters**				
Flowering time of the initial segment	F = 0.397, p = 0.533	F = 2.730, p = 0.081	F = 1.587, p = 0.221	Anova 2
Flowering time of the sympodial segment	W = 96, p = 0.059	SL F = 1.867, p = 0.189<br>SC F = 0.846, p = 0.450	NA	Wilcoxon test, Anova 1
Number total of inflorescences	F = 1.824, p = 0.195	F = 57.459, ***	F = 2.945, p = 0.080	Anova 2

% of aborted inflorescences on the main stem	F = 134.168, ***	F = 3.312, p = 0.051	F = 2.069, p = 0.144	Anova 2
Number of floral buds per inflo-rescence	W = 1072.5, *	SL F = 12.362, ***<br>SC F = 0.238, p = 0.811	NA	Wilcoxon test, Anova 1
% of open flowers per inflo-rescence	F = 0.228, p = 0.633	F = 9.092, ***	F = 0.020, p=0.980	Anova 2
Length of sepals (cm)	W = 1915.5, ***	SL F = 11.9, ***<br>SC F = 0.158, p = 0.854	NA	Wilcoxon test, Anova 1
Length of petals (cm)	F = 11.004, **	F = 0.030, p = 0.971	F = 2.908, p = 0.060	Anova 2
Length of stamens (cm)	F = 6.964, **	F = 0.787, p = 0.459	F = 4.762, *	Anova 2
Length of style + ovary (cm)	F = 99.580, ***	F = 4.347, *	F = 2.669, p = 0.075	Anova 2
Style exsertion (cm)	NA	SL NA<br>SC F = 2.188, p = 0.125	NA	Anova 1
Stigma receptivity (%)	W = 1172, ***	SL F = 0.391, p = 0.643<br>SC X^2 = 2.714, p = 0.257	NA	Wilcoxon test, Anova 1
Pollen viability (%)	W = 53157, ***	SL X^2 = 3.158, p = 0.206<br>SC F = 5.029, *	NA	Wilcoxon test, Kruskal-Wallis, Anova 1
Number of pollen grains per anther	F = 41.587, ***	F = 1.831, p = 0.170	F = 1.039, p = 0.361	Anova 2
**Elements concentrations**				
[Na] in inflorescences	F = 30.428, ***	F = 31.016, ***	F = 2.200, p = 0.120	Anova 2
[Na] in pericarp	F = 61.210, ***	F = 125.964, ***	F = 4.274, *	Anova 2
[Na] in seeds	t = -4.350, **	SL X^2 = 23.656, ***<br>SC F = 2.301, p = 0.119	NA	Student test, Kruskal-Wallis, Anova 1
[K] in inflorescences	F = 18.679, ***	F = 3.936, *	F = 1.622, p = 0.206	Anova 2
K/Na in inflorescences	W = 612, **	SL X^2 = 30.416, ***<br>SC F = 5.758, *	NA	Wilcoxon test, Kruskal-Wallis, Anova 1
[K] in pericarp	F = 0.431, p = 0.513	F = 21.136, ***	F = 1.971, p = 0.146	Anova 2
[K] in seeds	F = 9.615, **	F = 2.591, p = 0.082	F = 11.280, ***	Anova 2
[Ca] in inflorescences	W = 684, ***	SL F = 0.138, p = 0.883<br>SC F = 2.36, p = 0.099	NA	Wilcoxon test, Anova 1
[Ca] in pericarp	F = 112.806, ***	F = 4.810, *	F = 0.598, p = 0.552	Anova 2
[Ca] in seeds	F = 2.342, p = 0.131	F = 0.321, p = 0.727	F = 0.302, p = 0.741	Anova 2
[Mg] in inflorescences	F = 24.498, ***	F = 5.299, **	F = 2.538, p = 0.088	Anova 2
[Mg] in pericarp	F = 120.282, ***	F = 2.007, p = 0.142	F = 3.472, *	Anova 2
[Mg] in seeds	W = 815, ***	SL F = 3.043, p = 0.062<br>SC F = 4.040, *	NA	Wilcoxon test, Anova 1
**Expression of transporters**				
SOS1	F = 53.962, ***	F = 20.199, ***	F = 3.012, p = 0.056	Anova 2
SOS2	F = 3.046, p = 0.086	F = 13.799, ***	F = 5.235, **	Anova 2
SOS3	F = 24.908, ***	F = 19.928, ***	F = 2.640, p = 0.079	Anova 2
NHX3	F = 3.591, p = 0.063	F = 4.578, *	F = 1.540, p = 0.222	Anova 2
NHX4	F = 6.155, *	F = 3.867, *	F = 2.118, p = 0.129	Anova 2
HKT1;2	F = 7.290, **	F = 21.701, ***	F = 0.149, p = 0.862	Anova 2
SlHAK14	W = 342, ***	SL F = 4.479, *<br>SC F = 9.633, ***	NA	Wilcoxon test, Anova 1

SlAKT2/3	F = 0.018, p = 0.895	F = 9.824, ***	F = 1.249, p = 0.295	Anova 2
SlHAK3	F = 60.230, ***	F = 6.608, **	F = 2.827, p = 0.067	Anova 2
CNGC10	W = 656, p = 0.092	SL F = 0.488, p = 0.618<br>SC X^2 = 12.78, **	NA	Wilcoxon test, Anova 1, Kruskal-Wallis
**Fruits parameters**				
Fruit set (%)	W = 366, **	SL F = 0.515, p = 0.604<br>SC F = 0.077, p = 0.926	NA	Wilcoxon test, Anova 1
Number of seeds per fruit	W = 1313, ***	SL F = 4.969, *<br>SC X^2 = 3.750, p = 0.153	NA	Wilcoxon test, Anova 1
Number of seeds per gram of FW fruit	F = 717.138, ***	F = 0.971, p = 0.385	F = 5.720, **	Anova 2
Fresh weight (g)	W = 3224, ***	SL F = 69.022, ***<br>SC F = 2.417, p = 0.100	NA	Wilcoxon test, Anova 1
Dry weight (g)	W = 930, ***	SL F = 9.875, ***<br>SC F = 3.635, *	NA	Wilcoxon test, Anova 1
Water content (%)	W = 848, ***	SL X^2 = 848, ***<br>SC X^2 = 17.071, ***	NA	Wilcoxon test, Kruskal-Wallis
Circumference (cm)	F = 2072.392, ***	F = 33.195, ***	F = 52.463, ***	Anova 2
Sugar concentration (° Brix)	F = 174.027, ***	F = 4.411, *	F = 69.398, ***	Anova 2
pH	W = 581, **	SL F = 11.319, ***<br>SC F = 10.675, ***	NA	Wilcoxon test, Anova 1

FW, fresh weight, SC, *Solanum chilense,* SL, *Solanum lycopersicum,* DASt, days after stress imposition, NA, non-applicable
